# Coagulation dysfunction as an independent predictor of early pregnancy loss in women with polycystic ovary syndrome: a machine learning–based retrospective cohort study

**DOI:** 10.3389/fendo.2026.1846715

**Published:** 2026-07-22

**Authors:** Shengyun Yu, Zhicheng Wang, Xiuqi Yin, Qinhua Zhang

**Affiliations:** 1 Shuguang Hospital Affiliated to Shanghai University of Traditional Chinese Medicine, Shanghai, China; 2 Department of Traditional Chinese Medicine, Shanghai Huangpu District Cancer Prevention and Treatment Hospital (Hongshan Hospital), Shanghai, China

**Keywords:** adverse pregnancy outcomes, early pregnancy loss, coagulation dysfunction, hypercoagulable state, machine learning, polycystic ovary syndrome, predictive model

## Abstract

**Objective:**

Current studies on adverse pregnancy outcomes in polycystic ovary syndrome (PCOS) have primarily focused on metabolic and endocrine abnormalities, whereas systematic investigations addressing live birth outcomes and coagulation dysfunction remain limited. In addition, there is a lack of comprehensive tools for early pregnancy risk stratification. This study aimed to explore the associations between early pregnancy indicators and early pregnancy loss (EPL) in women with PCOS, identify key risk factors, and subsequently develop a clinically interpretable predictive model to facilitate early risk identification and individualized management.

**Methods:**

In this single-center retrospective cohort study, 2,290 women with PCOS who achieved natural singleton pregnancies were included. Participants were randomly divided into a training set (n=1,832) and a validation set (n=458). Demographic, endocrine-metabolic, and coagulation parameters were collected. Independent predictors were identified by multivariable logistic regression. Predictive models were constructed using logistic regression, random forest, and XGBoost algorithms, with model interpretability evaluated using Shapley Additive Explanations (SHAP). Furthermore, a temporally independent external cohort was introduced for external validation to assess the generalizability of the model.

**Results:**

Key predictors included D-dimer, fibrin degradation products (FDP), body mass index (BMI), anti-Müllerian hormone (AMH), and activated partial thromboplastin time (APTT). Elevated D-dimer and FDP levels were associated with increased EPL risk, suggesting enhanced coagulation activation and impaired microcirculation at the maternal–fetal interface. The XGBoost model showed the best performance, achieving an AUC of 0.926 (95% CI: 0.902–0.950). The model also maintained stable discriminative ability in temporal external validation (AUC = 0.705), although a certain degree of calibration drift was observed.

**Conclusion:**

Coagulation abnormalities during early pregnancy are closely associated with the occurrence of EPL in women with PCOS and may serve as independent risk factors. Machine learning models integrating multidimensional clinical indicators can effectively enable early identification of high-risk populations. Temporal external validation further suggested a certain degree of model generalizability, although additional optimization and validation are still required. These findings may provide valuable support for risk stratification and intervention decision-making during early pregnancy.

## 1 Introduction

Polycystic ovary syndrome (PCOS) is one of the most common endocrine disorders in women of reproductive age, and its detrimental impact on pregnancy outcomes has been well established (1, 2). Previous studies have reported that the incidence of early pregnancy loss (EPL) in women with PCOS ranges from 30% to 50%, which is significantly higher than that in the general population (3–5). Current explanations for this phenomenon primarily focus on hyperandrogenism, insulin resistance, dyslipidemia, and chronic low-grade inflammation. However, in clinical practice, these factors remain insufficient to fully account for the marked heterogeneity in pregnancy outcomes observed among women with PCOS (6, 7).

Clinical observations have indicated that a considerable proportion of women with PCOS exhibit abnormalities in coagulation-related parameters during early pregnancy, including elevated levels of fibrinogen, D-dimer, and plasminogen activator inhibitor-1 (PAI-1), suggesting a potential hypercoagulable state. Based on these findings, increasing attention has been directed toward alterations in the coagulation–fibrinolysis system in PCOS (8). However, the underlying nature of this hypercoagulability remains controversial. Some studies suggest that the prothrombotic tendency associated with PCOS can be largely explained by metabolic factors such as obesity, inflammation, and insulin resistance, rather than representing an intrinsic feature of PCOS (9, 10). In contrast, other evidence indicates that certain coagulation abnormalities may have independent pathological significance (8). This inconsistency has led to an unclear understanding of the pathophysiological role of hypercoagulability in PCOS (11).

In clinical practice, the assessment of coagulation function has predominantly focused on the mid-to-late stages of pregnancy or on populations with recurrent miscarriage, whereas relatively little attention has been paid to early pregnancy (8, 10). In fact, early pregnancy represents a critical window for embryo implantation and the establishment of endometrial receptivity. During this stage, alterations in uterine microcirculation and hemodynamics may directly influence implantation success. An imbalance in the coagulation–fibrinolysis system at this time could theoretically impair early embryonic development through microthrombus formation or insufficient perfusion 12); however, this mechanism has been insufficiently explored in previous studies.

On the other hand, previous studies on early pregnancy loss have predominantly focused on clinical miscarriage as the primary endpoint, with relatively limited attention given to biochemical pregnancy. Our observations indicate that biochemical pregnancy is relatively common among women with PCOS and may represent an earlier manifestation of implantation failure, warranting further investigation into its underlying mechanisms. Incorporating biochemical pregnancy and clinical miscarriage within a unified framework and analyzing them as sequential stages of early pregnancy loss may facilitate a more comprehensive understanding of the EPL process.

Based on these clinical observations and existing evidence, this study addresses a key question: whether coagulation abnormalities in early pregnancy remain independently associated with early pregnancy loss in women with PCOS after controlling for metabolic and endocrine factors. To this end, we conducted a single-center retrospective cohort study, systematically collecting coagulation, endocrine, and metabolic indicators during early pregnancy in women with PCOS, and assessed their independent effects using multivariable logistic regression analysis.

Furthermore, we employed machine learning approaches to integrate multidimensional clinical variables for predictive modeling, with the aim of assessing the individual-level predictive value of each factor and developing a clinically applicable risk assessment tool. Through these analyses, we sought to further elucidate the role of coagulation function in early pregnancy outcomes among women with PCOS based on real-world clinical data.

## 2 Patients and methods

### 2.1 Study design and patient selection

This study was a single-center retrospective cohort study involving pregnant women diagnosed with polycystic ovary syndrome (PCOS) at a tertiary hospital between October 2022 and December 2024. PCOS was diagnosed according to the Rotterdam criteria, defined by the presence of at least two of the following: ovulatory dysfunction, clinical or biochemical hyperandrogenism, and polycystic ovarian morphology. The study was approved by the institutional ethics committee, and all data were derived from anonymized clinical records.

The inclusion criteria were as follows:

(1) age between 20 and 45 years;
(2) diagnosis of PCOS according to the Rotterdam criteria;
(3) naturally conceived singleton pregnancy;
(4) complete follow-up data during early pregnancy (5–12 weeks of gestation);
(5) availability of clearly identifiable pregnancy outcomes, which could be definitively classified as normal pregnancy, biochemical pregnancy, clinical miscarriage, or other adverse pregnancy outcomes.

The exclusion criteria were as follows:

(1) multiple pregnancies or pregnancies achieved through assisted reproductive technologies;
(2) presence of severe systemic diseases that might affect pregnancy outcomes, including autoimmune diseases or severe hepatic, cardiac, or renal dysfunction;
(3) receipt of anticoagulant or antiplatelet therapy, including low-molecular-weight heparin or aspirin;
(4) missing key variables, defined as absence of core exposure variables or pregnancy outcome information that could substantially affect effect estimation;
(5) pregnancy outcomes that could not be clearly classified or showed overlapping classifications.

It should be noted that key variables primarily referred to core exposure and outcome-related indicators in this study, and their absence might have had a substantial impact on effect estimation; therefore, such cases were excluded. For missing data in certain covariates, including hormone levels and metabolic indicators, multiple imputation was subsequently applied to reduce sample size loss and potential bias. Ultimately, a total of 2,290 participants were included. The study was approved by the institutional ethics committee, and all data were obtained from anonymized clinical records.

### 2.2 Outcome definition

The primary outcome variable in this study was pregnancy outcome (Y), which was defined as an unordered multicategorical variable and classified into four groups according to follow-up results: normal pregnancy, biochemical pregnancy, spontaneous miscarriage, and other adverse pregnancy outcomes. All outcomes were determined based on outpatient and inpatient electronic medical records in combination with follow-up data.

The specific definitions were as follows:

(1) Normal pregnancy: pregnancy continuing beyond 12 weeks of gestation without documented early pregnancy loss or other adverse pregnancy events;
(2) Biochemical pregnancy: positive serum β-hCG detection reaching the pregnancy threshold without ultrasound-confirmed intrauterine gestational sac, followed by spontaneous decline in β-hCG levels;
(3) Spontaneous miscarriage: spontaneous pregnancy termination occurring within 12 weeks of gestation after ultrasound confirmation of an intrauterine pregnancy;
(4) Other adverse pregnancy outcomes: pregnancy-related adverse events not classified into the above three categories, mainly including hypertensive disorders of pregnancy (including gestational hypertension and preeclampsia), gestational diabetes mellitus (GDM), preterm birth (delivery at ≥28 and <37 weeks of gestation without overlap with miscarriage), fetal growth restriction (FGR), abnormal gestational weight gain (GWG), and venous thromboembolism (VTE). All outcomes were defined according to established clinical diagnostic criteria in conjunction with institutional follow-up records. The category of “other adverse pregnancy outcomes” included only event types that were actually observed during follow-up in this study.

It should be noted that biochemical pregnancy and spontaneous miscarriage were jointly defined as EPL and used as the primary outcome for predictive modeling. In contrast, “other adverse pregnancy outcomes” were incorporated as real-world background outcomes to reflect the overall distribution characteristics of pregnancy outcomes in women with PCOS. To minimize classification ambiguity, mutually exclusive definitions were applied across all outcome categories.

### 2.3 Clinical variables

Candidate variables collected in this study included demographic and clinical characteristics, previous pregnancy history, endocrine indicators, metabolic status, thyroid function, and coagulation–fibrinolysis system parameters. Specific variables included age, pre-pregnancy body mass index (BMI), history of adverse pregnancy outcomes, anti-Müllerian hormone (AMH), testosterone, insulin resistance, metabolic abnormalities, thyroid dysfunction, and coagulation-related indicators including thrombin time (TT), activated partial thromboplastin time (APTT), prothrombin activity, fibrinogen, D-dimer, antithrombin III (AT-III), fibrin degradation products (FDP), coagulation factor VIII, and anti-factor Xa activity. All variables were obtained from routine clinical records or laboratory test results. Detailed definitions, measurement units, variable types, and statistical handling methods for all candidate variables are provided in **Supplementary Table 1**.

Coagulation parameters were extracted from archived laboratory data obtained during the first clinical assessment in early pregnancy (5–12 weeks of gestation). Owing to the retrospective design of this study, the original database did not systematically record the exact gestational week at blood sampling. Given that gestational age is an important confounding factor affecting coagulation function, and precise temporal information was unavailable for adjustment, gestational age–based standardization, subgroup stratification, and restricted sensitivity analyses could not be performed.

To minimize the potential influence of medications on coagulation parameters, women with previous or current exposure to anticoagulant or antiplatelet therapy, including low-molecular-weight heparin and aspirin, were excluded during participant selection. Therefore, all coagulation-related laboratory parameters were collected in the absence of direct pharmacological interference and were considered to reflect the intrinsic coagulation status of the participants.

In addition, all anti-Müllerian hormone (AMH) measurements included in this study were derived from pre-pregnancy laboratory assessments performed within 12 months before conception. Given the relative short-term stability of AMH, this time window was considered to reasonably reflect ovarian functional status at the time of conception. All AMH data were retrospectively retrieved from medical records after pregnancy confirmation rather than obtained during pregnancy.

### 2.4 Data analysis and model development

Descriptive statistical analyses were initially performed to characterize the distributional features of the data, with appropriate statistical measures applied according to variable type. Missing data were subsequently handled using multiple imputation by chained equations (MICE) to reduce potential bias. After cohort cleaning, the outcome variable and core predictive factors, namely coagulation-related indicators, remained complete in the final analytical dataset. Missing values were confined to several covariates, including anti-Müllerian hormone (15.0%), testosterone (18.0%), thyroid dysfunction (10.4%), and metabolic abnormalities (17.0%). Given that the missing proportions for all variables were below 20%, 20 imputed datasets were generated. Predictive mean matching was applied for continuous variables, whereas logistic regression models were used for binary variables.

In the risk factor analysis, candidate variables associated with EPL were first screened through univariable analysis. Subsequently, these variables were incorporated into multivariable logistic regression models, with parameter estimation performed across the imputed datasets. Effect estimates were finally pooled according to Rubin’s rules to evaluate the independent effects of each variable after adjustment for metabolic, endocrine, and other potential confounding factors.

To objectively evaluate model performance, the entire cohort was randomly divided into a training set (n=1832) and an independent test set (n=458) at a ratio of 8:2. All models were developed in the training set and validated in the test set. For feature selection, the Boruta algorithm was initially applied to identify variables strongly associated with the outcome. Subsequently, least absolute shrinkage and selection operator (LASSO) regression was used for variable shrinkage and dimensionality reduction to improve model stability and reduce the risk of overfitting.

Based on the selected variables, three predictive models were constructed, including logistic regression (LR), random forest (RF), and extreme gradient boosting (XGBoost). Model performance was comprehensively evaluated from four aspects, including discrimination, calibration, clinical utility, and classification performance. Specifically, receiver operating characteristic (ROC) curves and the area under the curve (AUC), calibration curves, decision curve analysis (DCA), and confusion matrix–derived metrics including sensitivity, specificity, and F1 score were employed. In addition, Shapley Additive Explanations (SHAP) were applied to interpret the models, enabling quantification of the contribution of each variable to the prediction outcomes and thereby enhancing clinical interpretability.

Beyond the internal validation framework, an additional temporally independent validation cohort was constructed to further evaluate model generalizability. This cohort consisted of consecutively enrolled patients from a separate time period (January 2025 to June 2025), and none of the samples were involved in model development or internal validation. External validation was performed using the identical parameter settings and feature combinations established during model training, without any retraining or hyperparameter tuning, in order to avoid information leakage and preserve the independence of the validation process. Model discrimination (AUC), calibration (calibration intercept, calibration slope, and Brier score), and clinical utility (DCA) were systematically evaluated.

Given that EPL comprised both biochemical pregnancy and clinical miscarriage, which may represent different clinical stages of the early pregnancy loss process, additional analyses were further conducted. Using normal pregnancy as the reference category, separate multivariable logistic regression models were constructed for biochemical pregnancy and clinical miscarriage, respectively. XGBoost models were further applied to evaluate the discriminative performance for these two sub-outcomes. The results of these analyses are presented in the supplementary materials.

**Figure 1:**
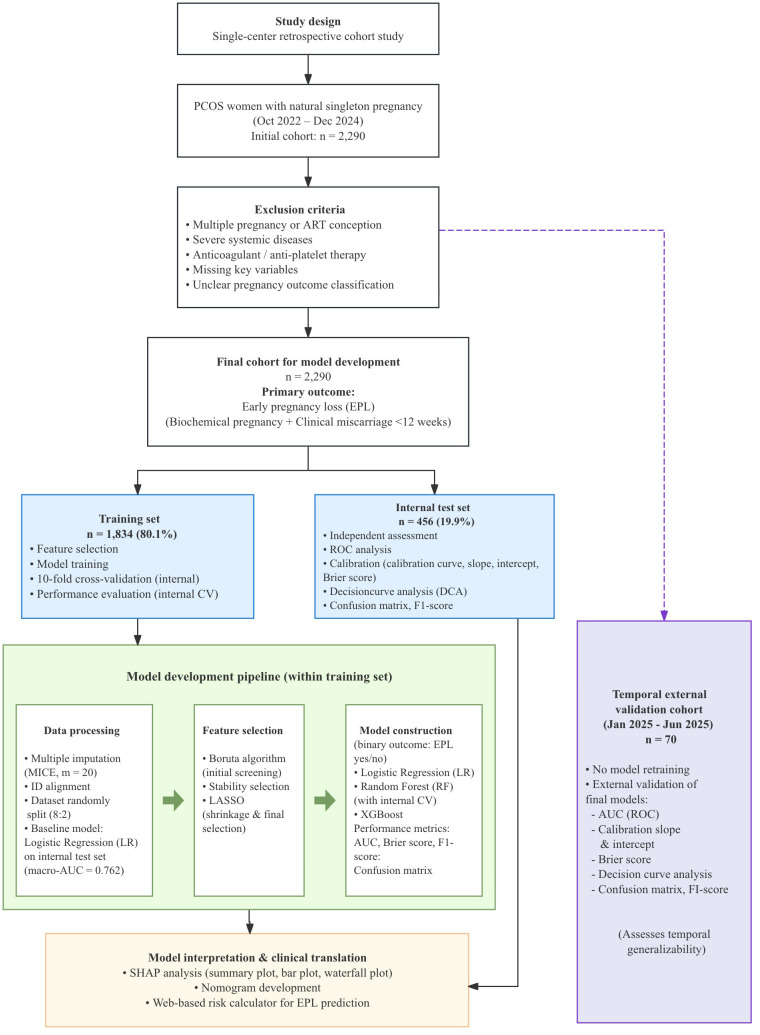
Study design and analytical workflow. This figure summarizes the study design and analytical workflow for predicting early pregnancy loss (EPL) in women with polycystic ovary syndrome (PCOS). This single-center retrospective cohort included 2,290 women with naturally conceived singleton pregnancies, randomly divided into a training set (n = 1,832) and an independent test set (n = 458) at an 8:2 ratio. All preprocessing and model development were performed in the training set, including multiple imputation by chained equations (MICE), feature standardization, and categorical encoding. Feature selection was conducted using the Boruta algorithm, stability selection, and LASSO regression. Logistic regression, random forest, and XGBoost models were developed based on selected features and validated in the test set. Model performance was assessed using ROC curves, calibration curves, decision curve analysis, and confusion matrix–based metrics. A temporally independent external cohort (January–June 2025) was used to evaluate generalizability. EPL was defined as biochemical pregnancy or clinical miscarriage before 12 weeks of gestation.

All statistical analyses were performed using SPSS software (version 27.0, USA) and R software (version 4.5.2, USA). All statistical tests were two-sided, and P < 0.05 was considered statistically significant. The overall study design and analytical workflow are illustrated in **Figure 1**.

## 3 Results

### 3.1 Characteristics of study participants

A total of 2,290 women with PCOS who achieved natural conception with singleton pregnancies were included in this study. Using stratified random sampling at an 8:2 ratio, 1,832 cases were assigned to the training set and 458 cases to the validation set. Baseline characteristics are presented in **Table 1**. Overall, the distributions of demographic characteristics, endocrine-metabolic parameters, and major coagulation-related indicators were comparable between the two datasets, indicating good baseline consistency.

**Table 1:** Baseline characteristics by pregnancy outcome (MI combined, m=20).

Variable	Other adverse outcome	Normal pregnancy	Biochemical pregnancy	Early abortion	Overall P	Effect size
Anti-Müllerian hormone, AMH (ng/mL)	7.21 (3.36, 9.16)	4.59 (2.03, 9.57)	6.47 (4.50, 8.45)	6.58 (5.13, 9.11)	<0.001***	0.022
Activated partial thromboplastin time, APTT (s)	27.63 (26.29, 29.81)	27.60 (25.76, 30.15)	29.50 (27.30, 36.20)	28.52 (25.50, 35.30)	<0.001***	0.02
Antithrombin III, AT-III (%)	95.30 (89.10, 103.50)	96.00 (89.40, 102.83)	97.40 (89.73, 104.50)	96.20 (88.88, 103.87)	1.0000	0
Age (years)	32.00 (29.00, 38.00)	32.00 (28.00, 36.00)	31.00 (27.00, 34.00)	31.00 (27.00, 35.00)	<0.001***	0.0052
Anti-Xa activity (IU/mL)	0.03 (0.00, 0.06)	0.03 (0.01, 0.06)	0.03 (0.00, 0.06)	0.03 (0.00, 0.06)	<0.001***	0.004
BMI (kg/m²)	22.86 (22.55, 23.20)	22.72 (22.11, 23.10)	23.01 (22.74, 23.28)	22.91 (22.65, 23.25)	<0.001***	0.049
D-dimer (µg/mL)	0.24 (0.16, 0.42)	0.30 (0.18, 0.48)	0.80 (0.46, 2.27)	0.70 (0.40, 0.91)	<0.001***	0.16
Fibrin degradation products, FDP (mg/L)	2.25 (0.90, 3.60)	3.05 (1.70, 5.54)	5.21 (1.70, 7.59)	4.71 (1.40, 6.90)	<0.001***	0.041
Fibrinogen, FIB (g/L)	2.95 (2.47, 3.43)	2.96 (2.53, 3.48)	3.28 (2.63, 4.33)	2.95 (2.49, 3.60)	<0.001***	0.006
Factor VIII (%)	112.10 (92.11, 131.47)	114.79 (96.42, 135.44)	131.20 (93.18, 221.70)	132.24 (100.30, 245.46)	<0.001***	0.027
Prothrombin activity (%)	105.60 (96.07, 112.85)	103.69 (94.25, 113.00)	104.77 (96.00, 116.61)	105.72 (96.00, 114.06)	<0.001***	0.001
Thrombin time, TT (s)	17.15 (16.40, 18.00)	17.20 (16.30, 18.30)	17.50 (16.60, 18.40)	17.30 (16.36, 18.03)	0.2737	0
Testosterone (nmol/L)	1.58 (1.18, 2.11)	1.48 (1.11, 1.91)	1.75 (1.26, 2.30)	1.63 (1.28, 2.17)	<0.001***	0.018
Insulin resistance						
Yes(1)	85 (45.0%)	587 (40.7%)	51 (34.2%)	157 (30.8%)		
Adverse obstetric history						
Yes(1)	70 (37.0%)	438 (30.4%)	50 (33.6%)	148 (29.0%)		
Dyslipidemia						
Yes(1)	69 (36.5%)	427 (29.6%)	32 (21.2%)	154 (30.1%)		
Thyroid dysfunction						
Yes(1)	20 (10.7%)	224 (15.5%)	29 (19.6%)	73 (14.2%)		

P values are Wald test results pooled across 20 imputations. *P<0.05, ^**^P<0.01, ^***^P<0.001.

Within the overall cohort, participants were categorized into four groups based on pregnancy outcomes: normal pregnancy (n=1,442), biochemical pregnancy (n=149), miscarriage (n=510), and other adverse pregnancy outcomes (n=189). Significant differences in multiple clinical and laboratory parameters were observed among these groups. For continuous variables, age, BMI, AMH, testosterone levels, and nearly all coagulation-related indicators, including D-dimer, FDP, APTT, fibrinogen, and coagulation factor VIII, differed significantly across the four groups (*p* < 0.001). Notably, D-dimer and FDP levels were markedly higher in the biochemical pregnancy and miscarriage groups compared with the normal pregnancy group, showing a clear increasing trend with worsening pregnancy outcomes.

For categorical variables, the prevalence of insulin resistance, metabolic abnormalities, and thyroid dysfunction also differed significantly among the outcome groups (*p* < 0.001). Furthermore, even in subgroups with comparable BMI and similar rates of metabolic abnormalities, coagulation parameters remained significantly different, suggesting that coagulation dysfunction may not be solely driven by metabolic status.

### 3.2 Identification of predictors

Early pregnancy loss (EPL) was defined as the primary outcome, referring to pregnancy termination within 12 weeks of gestation, including both biochemical pregnancy and clinical miscarriage. Candidate variables were selected not only based on statistical screening but also by integrating common clinical phenotypes of PCOS, routine laboratory parameters, and factors previously reported to be involved in PCOS pathophysiology (13). To enhance model stability and clinical interpretability, variables associated with EPL were first identified through univariate analysis and subsequently included in multivariable logistic regression models to assess their independent effects after adjusting for metabolic and endocrine confounders. Finally, Boruta and LASSO methods were applied to further refine the variable structure and mitigate multicollinearity.

Univariate logistic regression analysis demonstrated that multiple coagulation-related parameters were significantly associated with EPL, with particularly strong associations observed in biochemical pregnancy and miscarriage outcomes. In the biochemical pregnancy group, D-dimer, FDP, APTT, fibrinogen, and coagulation factor VIII were all significantly associated with pregnancy outcomes. The crude odds ratio (OR) for D-dimer was 1.62 (95% CI:1.47–1.78, p<0.001), and for FDP was 1.24 (95% CI:1.18–1.31, p<0.001). A similar pattern was observed in the miscarriage group, where D-dimer, FDP, APTT, and coagulation factor VIII were significantly associated with miscarriage risk; the crude OR for D-dimer was 1.37 (95% CI:1.25–1.49, p<0.001), and for FDP was 1.16 (95% CI:1.12–1.20, p<0.001). In addition, endocrine and metabolic indicators such as BMI, AMH, and testosterone were also associated with pregnancy outcomes (**Supplementary Table 2**).

In multivariable logistic regression analysis, coagulation-related indicators remained independently associated with EPL in both biochemical pregnancy and miscarriage groups. In the biochemical pregnancy group, D-dimer and FDP were identified as independent predictors, with adjusted ORs of 1.46 (95% CI:1.32–1.62, p<0.001) and 1.11 (95% CI:1.04–1.18, p<0.001), respectively. In the miscarriage group, D-dimer, FDP, APTT, and coagulation factor VIII also remained significant, with adjusted ORs of 1.23 (95% CI:1.12–1.35, p<0.001) for D-dimer and 1.13 (95% CI:1.09–1.18, p<0.001) for FDP. Notably, although BMI and testosterone levels were significantly associated with miscarriage, insulin resistance did not demonstrate an independent effect in the adjusted model. Overall, D-dimer and FDP emerged as the most predictive variables in this study, with consistently strong associations with increased EPL risk (**Table 2**).

**Table 2:** Multinomial logistic regression analysis of coagulation-related factors associated with pregnancy outcomes in women with polycystic ovary syndrome.

Variable	β	SE	aOR (95% CI)	P (Wald)
Biochemical pregnancy vs. normal pregnancy				
TT	0.034	0.053	1.03 (0.93–1.15)	0.524
APTT	0.076	0.016	1.08 (1.05–1.11)***	<0.001
PT	0.009	0.007	1.01 (1.00–1.02)	0.183
FIB	0.252	0.102	1.29 (1.05–1.57)*	0.014
DDimer	0.379	0.051	1.46 (1.32–1.62)***	<0.001
AT3	-0.008	0.008	0.99 (0.98–1.01)	0.299
FDP	0.116	0.031	1.12 (1.06–1.19)***	<0.001
FactorVIII	0.002	0.001	1.00 (1.00–1.00)*	0.015
Anti–Xa activity (rescaled)	-0.265	0.147	0.77 (0.57–1.02)	0.073
Early abortion vs. normal pregnancy				
TT	-0.065	0.036	0.94 (0.87–1.01)	0.071
APTT	0.061	0.010	1.06 (1.04–1.08)***	<0.001
PT	0.010	0.004	1.01 (1.00–1.02)*	0.011
FIB	-0.165	0.069	0.85 (0.74–0.97)*	0.017
DDimer	0.221	0.046	1.25 (1.14–1.37)***	<0.001
AT3	-0.002	0.005	1.00 (0.99–1.01)	0.687
FDP	0.133	0.021	1.14 (1.10–1.19)***	<0.001
FactorVIII	0.004	0.001	1.00 (1.00–1.01)***	<0.001
Anti–Xa activity (rescaled)	-0.464	0.122	0.63 (0.49–0.80)***	<0.001
Other adverse outcomes vs. normal pregnancy				
TT	-0.020	0.057	0.98 (0.88–1.10)	0.725
APTT	0.009	0.015	1.01 (0.98–1.04)	0.566
PT	-0.000228	0.006	1.00 (0.99–1.01)	0.969
FIB	-0.045	0.099	0.96 (0.79–1.16)	0.652
DDimer	0.120	0.085	1.13 (0.95–1.33)	0.158
AT3	0.008	0.007	1.01 (0.99–1.02)	0.255
FDP	-0.157	0.038	0.85 (0.79–0.92)***	<0.001
FactorVIII	-0.002	0.001	1.00 (1.00–1.00)	0.162
Anti–Xa activity (rescaled)	-0.333	0.245	0.72 (0.44–1.16)	0.174

aOR indicates adjusted odds ratio. 95% CI indicates 95% confidence interval. P values are Wald test results pooled across 20 imputations. Due to the extremely limited variability and high clustering of anti–factor Xa activity values in the study population, Anti–Xa activity was rescaled prior to model fitting to improve numerical stability and interpretability of regression estimates. This transformation did not alter the direction of association or statistical inference. *P<0.05, **P<0.01, ***P<0.001.

To further evaluate the heterogeneity within the composite endpoint of EPL, we performed supplementary modeling analyses stratified by biochemical pregnancy and clinical miscarriage. After multivariable adjustment, D-dimer and FDP remained independently associated with both outcomes. In the biochemical pregnancy model, the adjusted odds ratio (aOR) for D-dimer was 1.41 and for FDP was 1.10; in the clinical miscarriage model, the aOR for D-dimer was 1.21 and for FDP was 1.11. Further analysis using an XGBoost model demonstrated good discrimination for both subtypes, with an area under the receiver operating characteristic curve (AUC) of 0.9138 for biochemical pregnancy and 0.9392 for clinical miscarriage. Detailed results are provided in **Supplementary Figure 3** and **S4**.

### 3.3 Development and performance evaluation of predictive models

**Figure 2:**
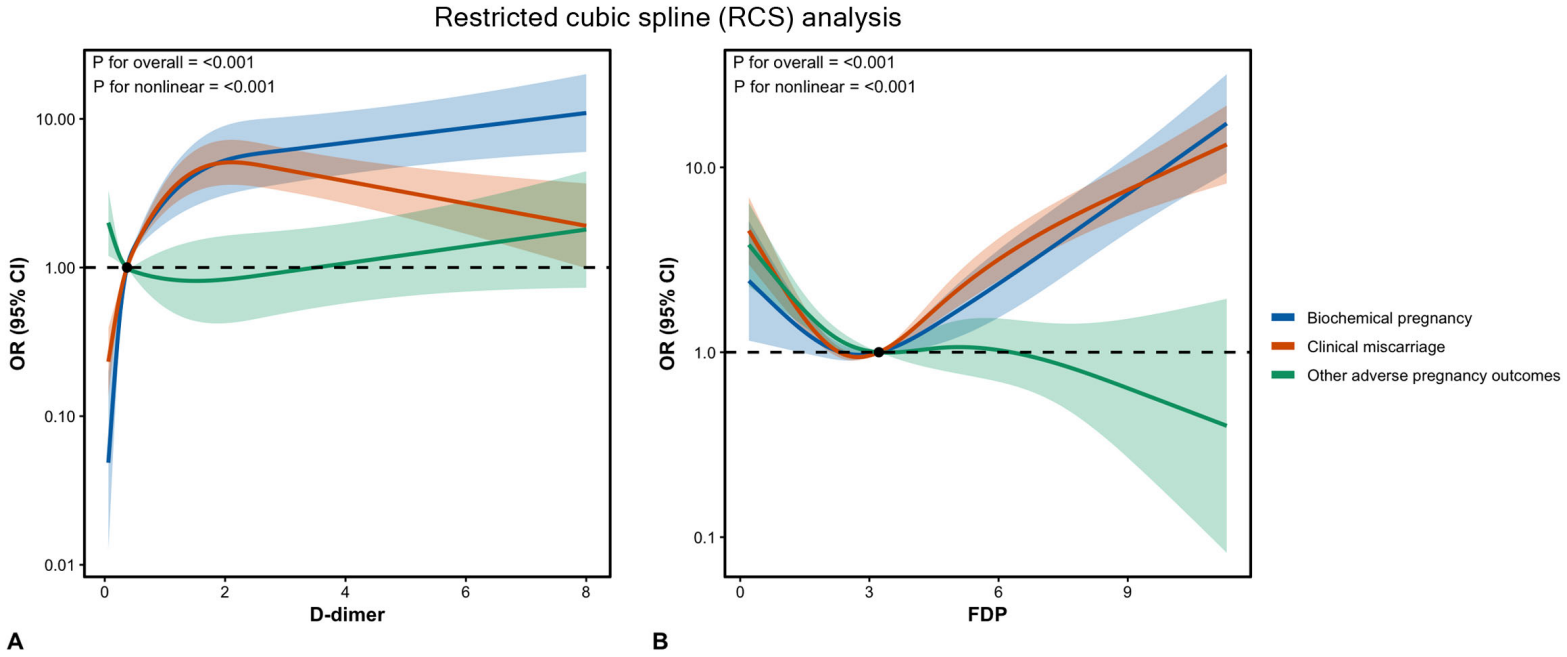
Restricted cubic spline analyses illustrating non-linear associations of D-dimer and fibrin degradation products with adverse pregnancy outcomes. **(A)** Restricted cubic spline curves depicting the association between D-dimer levels and odds ratios (ORs) for adverse pregnancy outcomes, including biochemical pregnancy, miscarriage, and other adverse outcomes, with normal pregnancy as the reference. **(B)** Restricted cubic spline curves illustrating the association between fibrin degradation products (FDP) and adverse pregnancy outcomes. Solid lines represent adjusted odds ratios derived from multinomial logistic regression models, and shaded areas indicate 95% confidence intervals. The horizontal dashed line denotes an OR of 1.0. P values for overall association and non-linearity were obtained from Wald tests.

Following variable selection, three predictive models — logistic regression (LR), random forest (RF), and extreme gradient boosting (XGBoost) — were constructed based on the final set of predictors. Model performance was evaluated using receiver operating characteristic (ROC) curves and the area under the curve (AUC). In the validation set, the XGBoost model demonstrated superior performance (AUC = 0.926), outperforming both the RF model (AUC = 0.903) and the LR model (AUC = 0.762), indicating a stronger discriminative ability of ensemble learning methods in this context (**Figure 2**). To further illustrate how the model interprets risk at the individual level, we presented representative cases to supplement the prediction results. In certain patients, despite none of the measured parameters exceeding conventional clinical intervention thresholds, concurrent borderline deviations across coagulation, metabolic, and endocrine profiles enabled the model to identify a potential high-risk status. These findings suggest that the model’s predictions do not rely on any single abnormal marker but instead integrate multiple parameters in combination to form a comprehensive risk assessment. Individual-level interpretability results are provided in the supplementary materials.

**Figure 3:**
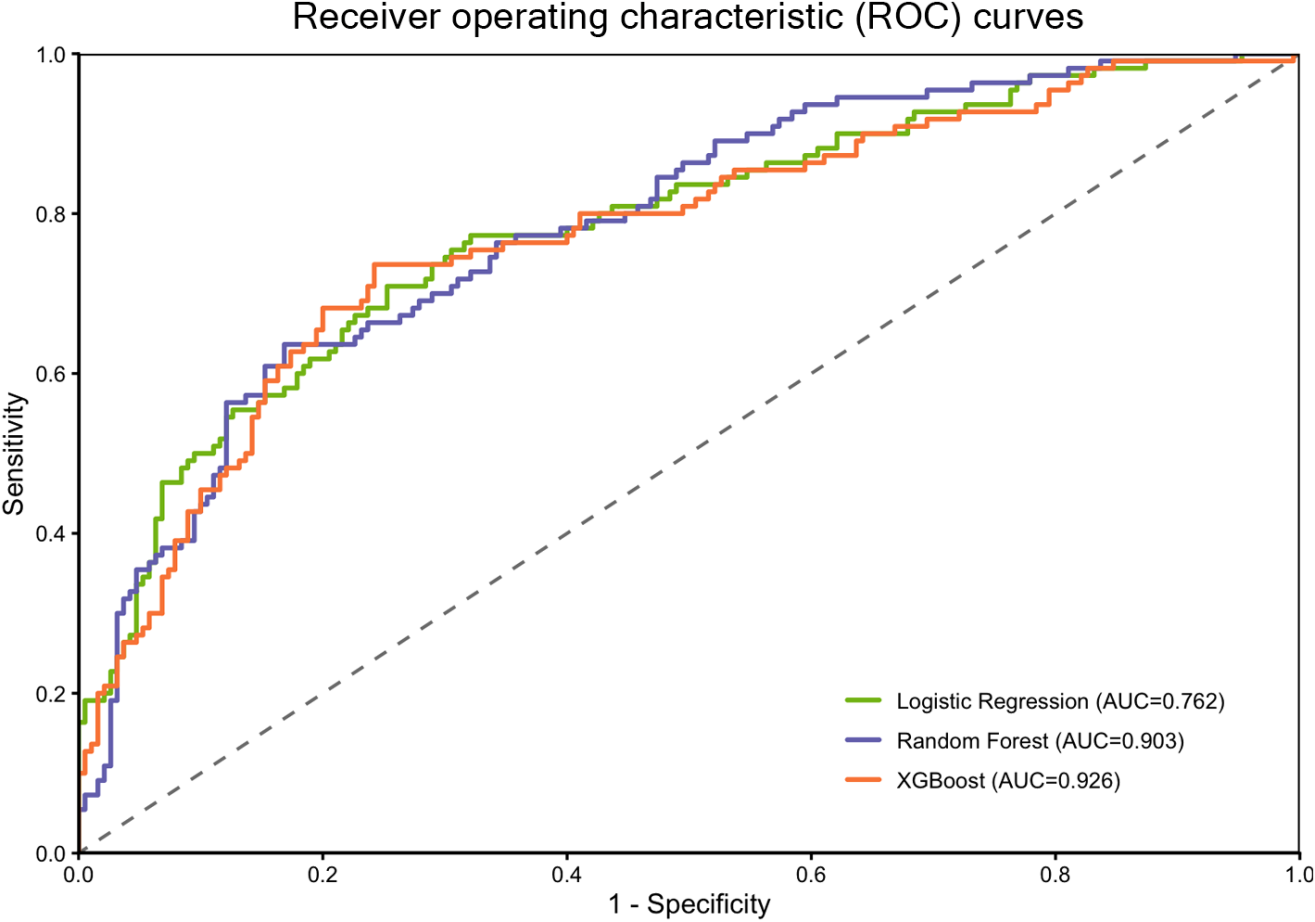
Receiver operating characteristic (ROC) curves comparing the discriminative performance of logistic regression, random forest, and XGBoost models for early pregnancy loss. ROC curves were generated based on predictions from the independent test set. All models were trained using identical training–testing splits and the same set of predictors to ensure fair comparison. Area under the curve (AUC) values reflect model discrimination only and do not capture calibration or clinical utility. The higher AUC observed for XGBoost indicates improved ranking of high-risk individuals but does not imply causal inference.

**Figure 4:**
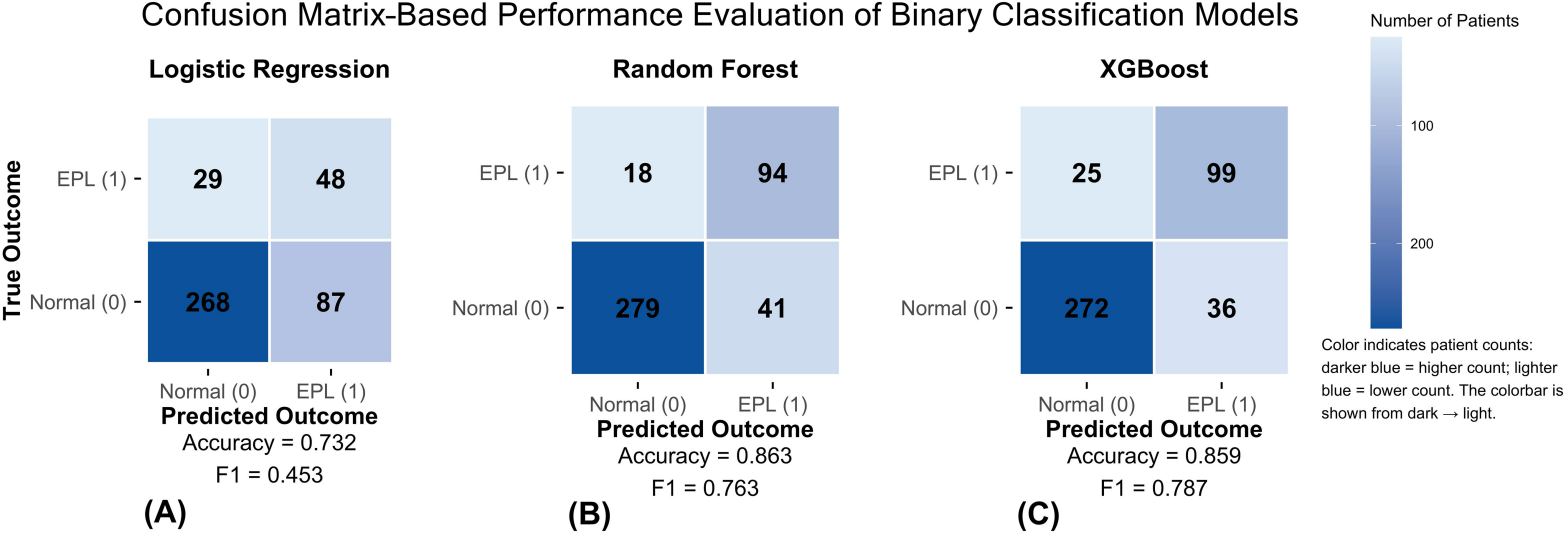
Confusion matrices comparing classification performance of logistic regression, random forest, and XGBoost models for early pregnancy loss. **(A)** Logistic regression model. **(B)** Random forest model. **(C)** XGBoost model. Classification performance was evaluated in the independent test set using thresholds optimized by the Youden index for each model. The logistic regression model demonstrated high sensitivity but low specificity, resulting in a high false-positive rate. In contrast, the random forest and XGBoost models achieved a more balanced trade-off between sensitivity and specificity, with substantially improved specificity and positive predictive value. Accuracy and F1 scores shown below each panel summarize overall classification performance.

**Figure 5:**
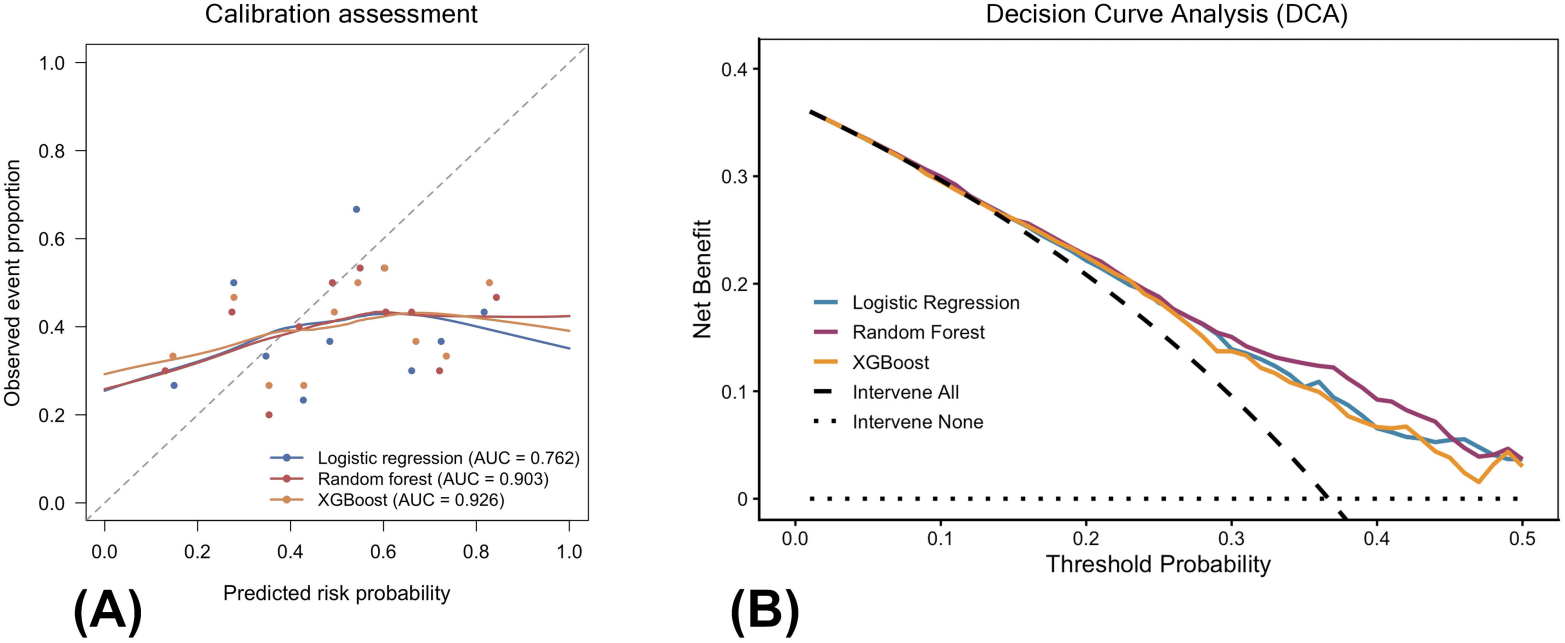
Calibration curves and decision curve analysis of logistic regression, random forest, and XGBoost models for early pregnancy loss. **(A)** Calibration performance reflects agreement between predicted and observed risks and does not assess discrimination. **(B)** Decision curve analysis evaluates clinical net benefit by simultaneously accounting for false-positive and false-negative classifications across threshold probabilities. All analyses were performed using the independent test set. Net benefit estimates should be interpreted in the context of risk stratification rather than diagnostic decision-making.

Further evaluation using calibration curves, decision curve analysis (DCA), and confusion matrices demonstrated that the XGBoost model not only exhibited superior discrimination but also showed good agreement between predicted probabilities and observed outcomes. Moreover, it provided greater net clinical benefit across a wide range of threshold probabilities, suggesting strong potential for clinical application (**Figures 3**, **4**, **5**).

**Figure 6:**
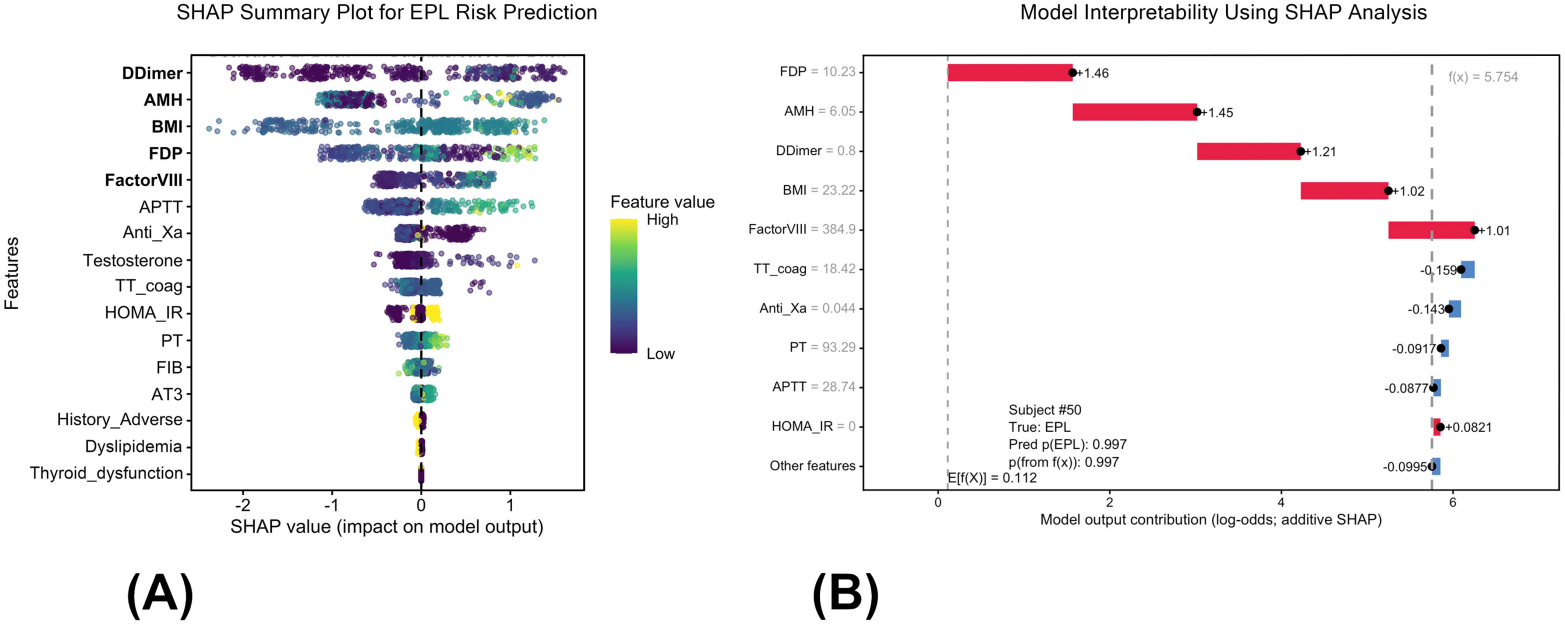
Global and case-specific interpretation of the XGBoost model using SHAP analysis for early pregnancy loss prediction. **(A)** SHAP summary plot showing the global importance and direction of effects of individual predictors in the XGBoost model for early pregnancy loss (EPL). Features are ranked by mean absolute SHAP values. Each point represents one individual in the independent test set, with color indicating the original feature value. **(B)** SHAP waterfall plot illustrating case-specific prediction for a representative high-risk individual who experienced EPL. The plot shows how individual feature contributions shift the model output from the average baseline risk to the final predicted risk.

**Figure 7:**
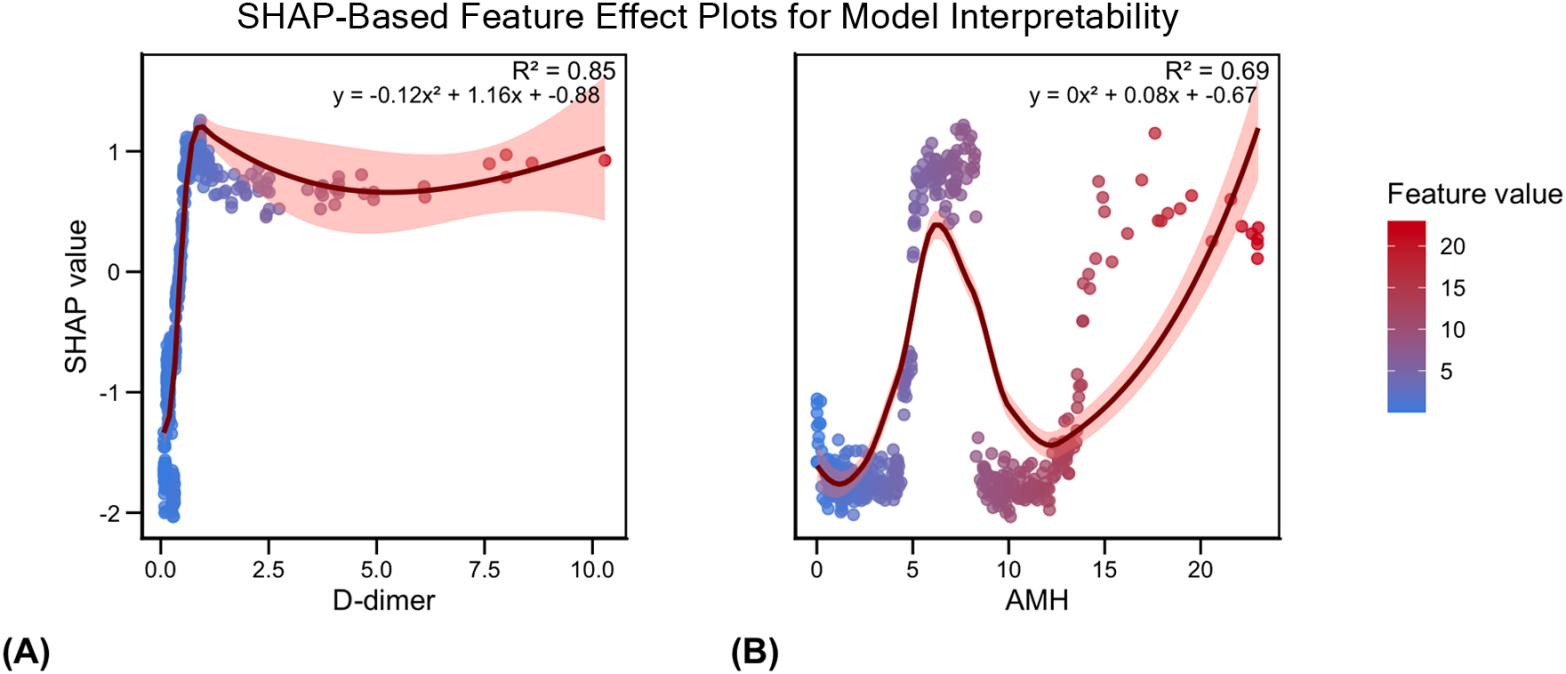
SHAP dependence plots illustrating the marginal effects of D-dimer and anti-Müllerian hormone on XGBoost model predictions for early pregnancy loss. SHAP values quantify the marginal contribution of each feature to the model’s predicted risk relative to the average prediction. Positive SHAP values indicate increased predicted risk, whereas negative values indicate decreased predicted risk. These plots describe model-learned associations and do not imply causal relationships or underlying biological mechanisms.

**Figure 8:**
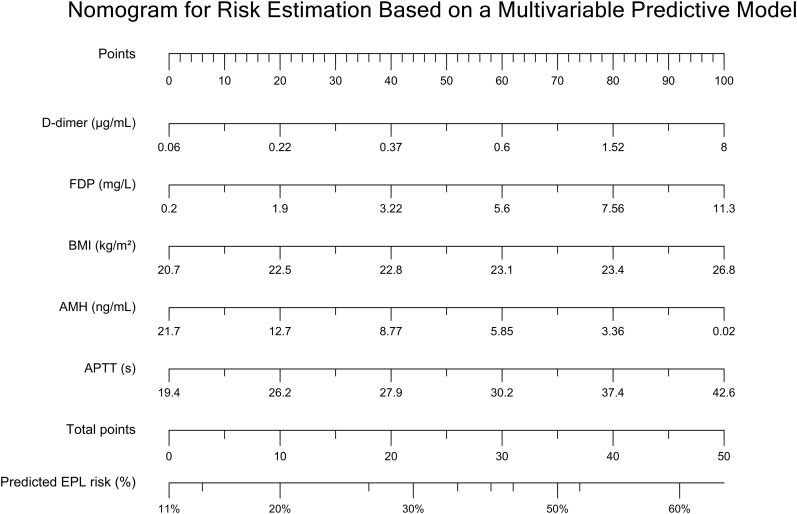
Nomogram for individualized prediction of early pregnancy loss risk in women with polycystic ovary syndrome. The nomogram was constructed based on the final optimized XGBoost model to facilitate individualized estimation of EPL risk in women with PCOS. Five predictors were incorporated, including D-dimer, fibrin degradation products (FDP), body mass index (BMI), anti-Müllerian hormone (AMH), and activated partial thromboplastin time (APTT).For each predictor, a corresponding point value is assigned by projecting the patient’s value to the top points scale. The total points are obtained by summing the individual scores and are then mapped to the predicted probability of EPL shown on the bottom axis. The AMH axis is reversed to reflect its protective association with EPL risk, such that higher AMH values correspond to lower point assignments.

In addition, SHAP analysis further confirmed the interpretability of the model (**Figure 6**, **7**). The results indicated that D-dimer, FDP, and other included variables contributed substantially to the model predictions, consistent with the key predictors identified in the regression analyses. Based on the XGBoost model, a nomogram was constructed (**Figure 8**), and an online risk calculator was further developed to facilitate individualized assessment of EPL risk in women with PCOS in clinical practice.

To address the potential impact of outcome imbalance, we determined the optimal classification threshold in the independent test set using Youden’s index and conducted additional analyses of model performance. The results showed that, without resampling or weighting, the logistic regression model maintained a relatively balanced discriminative performance (sensitivity 0.706, specificity 0.735, F1 score 0.588, balanced accuracy 0.721), indicating no marked bias toward either EPL or non-EPL individuals. Examination of the confusion matrices revealed that both random forest and XGBoost models preserved high sensitivity while markedly reducing the false-positive rate, thereby improving specificity and positive predictive value—an improvement with direct clinical relevance for minimizing misclassification of normal pregnancies. Furthermore, calibration analysis conducted in the primary evaluation demonstrated that predicted probabilities from the logistic regression model were largely consistent with observed outcomes. In comparison, XGBoost exhibited more stable performance in balancing discrimination and calibration, supporting its suitability for the current dataset structure.

### 3.4 External validation

To further evaluate the generalizability of the model, an external validation analysis was conducted using a temporally independent cohort comprising 70 patients, including 33 cases (47.1%) of EPL and 37 cases (52.9%) of ongoing normal pregnancy. In terms of discrimination, the XGBoost model achieved an area under the receiver operating characteristic curve (AUC) of 0.705 in the external validation set (**Supplementary Figure 1**), indicating that the model retained moderate discriminative ability in independent data, albeit with some reduction compared with the internal test set. Regarding calibration, the calibration intercept was 0.18, the calibration slope was 0.62, and the Brier score was 0.229. While the XGBoost model demonstrated good discrimination and acceptable calibration in the internal test set, a reduced calibration slope was observed in the temporal validation cohort, suggesting possible overfitting. Decision curve analysis (**Supplementary Figure 2**) showed that, within a threshold probability range of approximately 0.35–0.40, the model provided a positive net benefit compared with both “treat-all” and “treat-none” strategies.

Overall, although the model’s performance declined somewhat in external validation, it maintained stable discriminative capacity and retained clinically relevant utility.

## 4 Discussion

### 4.1 Main results

An increased risk of adverse pregnancy outcomes in women with PCOS has been consistently demonstrated in previous studies, among which early pregnancy loss (EPL) is particularly common, with an incidence significantly higher than that in the general population (3, 4). Previous investigations have primarily attributed this phenomenon to hyperandrogenism, insulin resistance, obesity, and metabolic disturbances (6, 7). However, these factors alone remain insufficient to fully explain the marked clinical heterogeneity in pregnancy outcomes observed among women with PCOS. In the present study, we approached this issue from the perspective of coagulation–fibrinolysis status during early pregnancy and found that both D-dimer and fibrin degradation products (FDP) were significantly elevated in patients with biochemical pregnancy and clinical miscarriage. Importantly, these associations remained independent after adjustment for BMI as well as endocrine and metabolic factors. These findings suggest that coagulation abnormalities may not merely represent secondary consequences of metabolic or endocrine dysfunction, but rather constitute an independent pathological dimension underlying the risk of EPL in women with PCOS.

Further analyses demonstrated that D-dimer and FDP were the most robust coagulation-related variables identified in this study. In the biochemical pregnancy model, the adjusted ORs were 1.41 for D-dimer and 1.10 for FDP, whereas in the clinical miscarriage model, the adjusted ORs were 1.21 and 1.11, respectively. In addition, the XGBoost models for both subtypes of EPL exhibited excellent discriminative performance, with AUC values of 0.9138 for biochemical pregnancy and 0.9392 for clinical miscarriage. These findings indicate that abnormalities in the coagulation–fibrinolysis system may not be restricted to a specific stage of pregnancy failure, but instead may exert a persistent influence across the continuum from implantation maintenance to the establishment of early clinical pregnancy.

In addition to D-dimer and FDP, APTT, BMI, and AMH were also retained in the final predictive model. The inclusion of APTT suggests that the risk of EPL may involve not only elevated fibrin degradation activity, but also alterations in the intrinsic coagulation pathway. BMI reflects the metabolic burden, inflammatory amplification, insulin resistance, and endothelial dysfunction commonly present in women with PCOS. Meanwhile, AMH may be better interpreted as a marker of ovarian phenotypic severity in PCOS rather than merely an indicator of ovarian reserve. Collectively, these findings indicate that the risk of EPL in pregnant women with PCOS is not determined by a single abnormal parameter, but rather arises from the combined effects of coagulation dysfunction, metabolic disturbance, and endocrine imbalance.

### 4.2 Significance of coagulation abnormalities in EPL assessment in women with PCOS

Increasing attention has been directed toward the prothrombotic tendency observed in women with PCOS. Hyperandrogenism, insulin resistance, and chronic low-grade inflammation may disrupt the balance of the coagulation–anticoagulation–fibrinolysis system by influencing coagulation factor expression, platelet activity, and fibrinolytic function (9). Previous studies have demonstrated that women with PCOS frequently exhibit elevated fibrinogen and plasminogen activator inhibitor-1 (PAI-1) levels, thereby maintaining a persistent hypercoagulable state (8). In parallel, endothelial dysfunction may further increase the propensity for thrombosis and venous thromboembolism (VTE) (14, 15). These pathophysiological alterations are considered potential contributors to the development of spontaneous pregnancy loss in PCOS.

However, in current clinical practice, attention to coagulation function is mainly focused on recurrent miscarriage, placental-related complications during mid-to-late pregnancy, or populations with established thrombotic risk, whereas insufficient emphasis has been placed on coagulation status during early pregnancy in women with PCOS (8, 10). Early pregnancy represents a critical period for embryo implantation, establishment of endometrial receptivity, decidualization, and microcirculatory remodeling at the maternal–fetal interface. If coagulation activation or fibrinolytic imbalance is already present at this stage, even without reaching conventional clinical intervention thresholds, it may still impair embryonic development through microthrombus formation, inadequate perfusion, or local microcirculatory instability (12). Therefore, our findings suggest that even mild abnormalities or borderline shifts in coagulation-related indicators during early pregnancy may carry potential value for risk stratification in pregnant women with PCOS.

### 4.3 Possible mechanistic explanations

Among the specific markers, D-dimer, a sensitive indicator of fibrin degradation and thrombotic activity, reflects ongoing coagulation and secondary fibrinolysis when elevated. During pregnancy, a moderate level of coagulation activity is necessary to maintain placental perfusion; however, excessive activation may result in microthrombus formation and impaired placental blood flow (16, 17). Previous studies have also linked elevated D-dimer levels to adverse pregnancy outcomes, including miscarriage and placental dysfunction (18). The present study further demonstrates that, even after adjusting for metabolic factors, D-dimer remains significantly associated with EPL risk in women with PCOS, suggesting a potential role in embryo implantation and the maintenance of early pregnancy.

FDP, as the terminal product of fibrin degradation, reflects activation of the fibrinolytic system when elevated. Increased FDP levels typically indicate a persistent imbalance between coagulation and fibrinolysis, which may destabilize local microcirculation in the endometrium and thereby impair embryo implantation. In the present study, FDP maintained a stable positive association across both biochemical pregnancy and clinical miscarriage outcomes, further supporting the potential involvement of coagulation–fibrinolysis disturbances in the development of EPL.

APTT also demonstrated independent predictive value in this study, suggesting that EPL risk is not solely reflected by elevated fibrin degradation products but also involves alterations in the overall dynamics of the intrinsic coagulation pathway. APTT represents the integrated process of the intrinsic coagulation cascade from contact activation to fibrin formation and is influenced by multiple coagulation factors and inhibitors. Therefore, its clinical significance extends beyond the traditional assessment of bleeding risk; in pregnancy-related contexts, APTT changes may reflect coagulation dynamics under hypercoagulable conditions (19). Previous studies have suggested that APTT may serve as an indicator of miscarriage risk (20), and patients with recurrent miscarriage may exhibit elevated factor VIII levels and shortened APTT, indicating enhanced intrinsic coagulation activation (21). Accordingly, the value of APTT in the present study may be better interpreted as complementary evidence of coagulation network disturbance rather than as an isolated core determinant of EPL. When analyzed together with D-dimer and FDP, APTT provides a more comprehensive characterization of the multilayered abnormalities extending from coagulation activation and fibrin turnover to intrinsic pathway imbalance during early pregnancy in women with PCOS.

The inclusion of BMI in the predictive model also has a clear pathological basis. In women with PCOS, BMI represents not merely body habitus but an integrated indicator of metabolic burden, inflammatory status, insulin resistance, and endothelial dysfunction. Meta-analyses have demonstrated that pre-pregnancy overweight or obesity significantly increases the risk of miscarriage and other adverse pregnancy outcomes in PCOS (22). Meanwhile, studies investigating the hypercoagulable state in PCOS have suggested that some coagulation abnormalities may be partially explained by BMI, inflammation, and insulin resistance, indicating that obesity is not simply a background characteristic but an active contributor to pathological amplification. Expansion of adipose tissue may promote chronic low-grade inflammation, enhance endothelial activation and oxidative stress, and aggravate fibrinolytic suppression through worsening insulin resistance. During early pregnancy, establishment of decidual perfusion depends on finely regulated vascular remodeling. When obesity-related hypercoagulability, inflammation, and metabolic disturbances coexist, the maternal–fetal interface may become more susceptible to microthrombus formation, unstable perfusion, and impaired nutrient exchange efficiency (10).

Interpretation of AMH should likewise avoid oversimplification. Secreted by granulosa cells of small antral follicles, AMH is frequently persistently elevated in women with PCOS and is closely associated with increased antral follicle count, the severity of ovulatory dysfunction, and overall phenotypic burden of the syndrome (23). Therefore, in the present study, AMH is more appropriately interpreted as a marker of ovarian phenotypic severity in PCOS rather than merely an indicator of ovarian reserve. Previous studies investigating the relationship between AMH and miscarriage risk have yielded inconsistent findings. Schumacher et al. reported an inverse association between AMH levels and miscarriage risk in naturally conceived pregnancies; however, their study excluded patients with PCOS and endometriosis and primarily focused on the influence of ovarian reserve on fertility outcomes (24). By contrast, Arkfeld et al., based on a large ART database, demonstrated that the predictive value of AMH for miscarriage was more evident in infertile women without PCOS, whereas its predictive performance was attenuated in the PCOS population. The authors proposed that elevated AMH levels in PCOS may reflect follicular arrest and phenotypic severity rather than embryo quality itself (25). Accordingly, the inclusion of AMH in the present model may be more reasonably interpreted as supplementing endocrine and ovarian phenotypic information specific to PCOS, thereby allowing risk assessment to simultaneously capture ovarian phenotype and coagulation-related abnormalities.

### 4.4 Model interpretation and clinical translation

With respect to model construction, this study extended beyond conventional logistic regression by incorporating random forest and XGBoost to integrate coagulation, metabolic, and endocrine variables into a unified analytical framework. In the independent test set, the XGBoost model achieved the best performance, with an AUC of 0.926, outperforming both the random forest model (AUC = 0.903) and the logistic regression model (AUC = 0.762). These findings suggest that nonlinear models may be better suited for capturing the multifactorial interactions and complex inter-variable relationships underlying pregnancy outcomes in women with PCOS.

To address the potential influence of outcome imbalance, the optimal classification threshold was determined in the independent test set based on the Youden index, followed by further evaluation of classification performance. Without applying resampling or class-weighting strategies, the logistic regression model still maintained relatively balanced discrimination, with a sensitivity of 0.706, specificity of 0.735, F1 score of 0.588, and balanced accuracy of 0.721. Compared with logistic regression, the random forest and XGBoost models maintained relatively high sensitivity while reducing the false-positive rate and improving specificity as well as positive predictive value. This improvement may have practical clinical relevance in reducing the misclassification of normal pregnancies as high-risk cases.

It should be emphasized that the predictions generated by the XGBoost model cannot be interpreted using fixed regression coefficients in the same manner as conventional logistic regression models. The contribution of each variable within the model does not represent a constant weight; rather, risk estimation is jointly determined through nonlinear interactions among different combinations of individual features. Therefore, SHAP analysis was employed to improve model interpretability. The SHAP results demonstrated that D-dimer, AMH, BMI, FDP, and APTT were the core variables with the greatest contributions to the model output. Among them, D-dimer showed the highest mean absolute SHAP value, followed by AMH and BMI, whereas FDP and APTT also exhibited substantial contributions. Importantly, this ranking was highly consistent with the results obtained from Boruta-LASSO feature selection and multivariable logistic regression analyses, suggesting that the model predictions were not driven by isolated outliers or statistical fluctuations, but rather by clinically meaningful combinations of variables.

With regard to the directionality of variable effects, elevated D-dimer, FDP, and BMI levels were consistently associated with increased EPL risk. By contrast, AMH primarily functioned in the model as a supplementary indicator reflecting ovarian phenotypic characteristics in PCOS. Its predictive direction differed from that of the coagulation-related markers, suggesting that its predictive significance should not be directly equated with a pathogenic effect. In other words, the present model identifies a multidimensional risk structure jointly shaped by coagulation abnormalities, metabolic background, and ovarian phenotypic features, rather than a single linear causal pathway.

Based on the XGBoost model, a nomogram was constructed, and an online risk calculator (https://pcos-epl.onrender.com) was further developed. This tool integrates multiple indicators, including D-dimer, FDP, BMI, AMH, and APTT, into an individualized probability estimate of EPL risk. It may be particularly useful for identifying high-risk individuals in whom several indicators simultaneously remain within a borderline range. In such patients, individual variables may not reach conventional clinical intervention thresholds; however, the overall risk may still increase when coagulation, metabolic, and endocrine parameters exhibit concurrent borderline deviations. Individual-level SHAP analysis further suggested that some patients may still present a high-risk profile despite only mild abnormalities or borderline values across individual indicators, potentially because of cumulative or counterbalancing effects among multiple variables. This pattern may be easily overlooked in traditional threshold-based assessment frameworks.

Considering that digital prediction tools may not always be readily accessible in frontline clinical settings, we further simplified the core variables into a bedside risk stratification framework. Rather than providing an exact numerical risk estimate, this framework categorizes patients into low-, intermediate-, and high-risk groups based on the combined dimensions of coagulation status, metabolic burden, and endocrine background. Patients were considered low risk when coagulation-related indicators (D-dimer, FDP, and APTT) showed no evident abnormalities and when BMI and AMH did not suggest substantial metabolic or ovarian phenotypic burden. Intermediate risk was defined by the presence of a markedly abnormal single indicator or borderline elevation across multiple variables. High risk was defined by concurrent coagulation activation, particularly simultaneous elevation of D-dimer and FDP, combined with elevated BMI or abnormal AMH levels. This stratification strategy emphasizes recognition of the cumulative effect of multiple borderline abnormalities and may assist clinicians in rapidly determining follow-up intensity in the absence of computational tools.

Nevertheless, both the predictive model and the stratification framework should be regarded as tools for risk identification and follow-up support rather than direct treatment decision-making instruments. At present, periconceptional management in women with PCOS remains primarily focused on metabolic and endocrine regulation, whereas assessment of coagulation function is relatively underemphasized. Moreover, several commonly used therapeutic interventions may themselves influence thrombotic risk. For example, combined oral contraceptives (COCs), although effective in improving hyperandrogenic manifestations, may increase the risk of thrombosis because of their estrogen component (26). Ovulation induction therapy may increase the risk of ovarian hyperstimulation syndrome and multiple pregnancy, both of which are closely associated with hypercoagulability (27). In selected high-risk populations, low-molecular-weight heparin and low-dose aspirin have been used to improve pregnancy outcomes, although their indications and optimal timing remain controversial (28, 29). Metformin, while improving insulin resistance, may also indirectly influence the coagulation system through modulation of inflammatory and metabolic status (30). Lifestyle interventions, including weight control and exercise, as first-line management strategies for PCOS, may likewise indirectly affect coagulation function through metabolic improvement. Taken together, the present findings support increased attention to coagulation abnormalities during early pregnancy risk assessment in women with PCOS; however, they should not be directly interpreted as evidence supporting anticoagulant or antiplatelet treatment benefit.

### 4.5 Limitations and perspectives

Several limitations of this study should be acknowledged. First, this was a single-center retrospective cohort study. Although the sample size was relatively large, the inclusion and exclusion process may still have introduced selection bias. For example, exclusion of individuals with missing key variables may have resulted in a study population biased toward patients with better follow-up adherence or more complete laboratory records.

To further evaluate model generalizability, an additional temporally independent validation cohort was included, comprising 70 patients, including 33 EPL cases (47.1%) and 37 normal pregnancies (52.9%). In this cohort, the XGBoost model achieved an AUC of 0.705, indicating that the model retained moderate discriminative ability in an independent temporal cohort, although the performance decreased compared with the internal test set. In terms of calibration, the calibration intercept was 0.18, the calibration slope was 0.62, and the Brier score was 0.229, suggesting a certain degree of calibration drift and potential overfitting. Decision curve analysis further demonstrated that the model still provided positive net benefit compared with the “treat-all” and “treat-none” strategies within a threshold probability range of approximately 0.35–0.40.

However, given the limited sample size of the validation cohort and its derivation from the same center, future multicenter studies with larger sample sizes and prospective external validation are still required.

Second, this study was limited by the laboratory assessment time window. Coagulation-related indicators were collected during the first clinical evaluation in early pregnancy between 5 and 12 weeks of gestation. However, because of the retrospective nature of the database, the exact gestational week on the day of blood sampling was not consistently recorded.

Given that coagulation function dynamically changes across gestation, the absence of precise sampling-time information limited gestational age–based standardization, subgroup stratification, and restricted sensitivity analyses. Therefore, the present findings should be interpreted as reflecting the association between coagulation status at the initial early-pregnancy clinical assessment and pregnancy outcomes, rather than the longitudinal trajectory of coagulation markers across gestation. Accordingly, causal inference cannot be established based on the current data.

Third, residual confounding related to medication exposure may still exist. To minimize the direct influence of anticoagulant or antiplatelet therapies on coagulation-related indicators, patients receiving treatments such as low-molecular-weight heparin or aspirin were excluded during participant selection. Therefore, the coagulation parameters analyzed in this study primarily reflected endogenous coagulation characteristics in the absence of direct pharmacological intervention by these agents.

However, metformin exposure could not be further quantified in the present study. Metformin is commonly used in women with PCOS and may indirectly influence coagulation function and pregnancy outcomes through improvement of insulin resistance and inflammatory status. Nevertheless, in the retrospective database, records regarding treatment duration, dosage, adherence, and drug discontinuation were incomplete. In addition, some patients exhibited intermittent medication use because of gastrointestinal intolerance, resulting in substantial heterogeneity in exposure definition. Under these circumstances, incorporating metformin simply as a binary variable might instead introduce misclassification bias.

Therefore, insulin resistance status was retained as a marker of metabolic background, whereas metformin exposure was not included as an independent predictive variable in the present analysis. Future prospective studies should incorporate standardized recording of medication exposure windows, dosage, and treatment adherence.

Finally, this study did not directly verify the pathological link between coagulation abnormalities and microcirculatory alterations at the maternal–fetal interface. Although D-dimer, FDP, and APTT can reflect systemic coagulation–fibrinolysis status, they cannot directly demonstrate the presence of local microthrombus formation, perfusion impairment, or disrupted nutrient exchange within the endometrium or early placenta.

Therefore, future studies should incorporate targeted sampling within clearly defined gestational windows and combine placental pathology, endothelial function markers, and molecular biological investigations to further elucidate the specific pathways through which coagulation abnormalities contribute to early pregnancy loss in women with PCOS.

## 5 Conclusion

In summary, the present study demonstrated that abnormalities in the coagulation–fibrinolysis system during early pregnancy in women with PCOS, particularly elevated D-dimer and FDP levels, were consistently associated with the occurrence of EPL. After integrating coagulation-related indicators with variables including BMI, AMH, and APTT, the XGBoost model achieved strong discriminative performance in the internal test set (AUC = 0.926) and retained moderate discriminative ability in a temporally independent validation cohort (AUC = 0.705).

The online risk calculator (https://pcos-epl.onrender.com) and the bedside stratification framework may serve as practical tools for early risk identification and follow-up support. However, the model output represents only a statistical risk estimate and should neither replace clinical judgment nor be directly used as a basis for initiating anticoagulant or antiplatelet therapy. Future multicenter prospective studies are still required to further validate the stability of the model and to determine whether coagulation abnormalities during early pregnancy in women with PCOS may represent clinically actionable targets for intervention.

## Data Availability

The datasets presented in this article are not readily available due to patient confidentiality constraints. Requests to access the datasets should be directed to Shengyun Yu, 1099035054@qq.com.
