# Supplementary material for: Coagulation dysfunction as an independent predictor of early pregnancy loss in women with polycystic ovary syndrome: a machine learning–based retrospective cohort study
Source: Front Endocrinol (Lausanne). 2026 Jul 22;17:1846715. doi: 10.3389/fendo.2026.1846715 (PMC13437431; doi:10.3389/fendo.2026.1846715)
Supplement: Supplementary file 1 [file DataSheet1.docx]

**Supplementary Information (SI)**

A. **Supplementary notes**

**Notes S1.** Web-based EPL Risk Calculator

1. Overview

A web-based Early Pregnancy Loss (EPL) risk calculator was developed based on the final optimized machine-learning model derived from this study. The calculator is designed to facilitate individualized risk estimation of EPL among women with polycystic ovary syndrome (PCOS) using a limited number of routinely available clinical and laboratory variables.

The calculator is publicly accessible at: *<https://pcos-epl.onrender.com/>*

2. Target Population and Outcome

The calculator is intended for use in women with PCOS who are planning pregnancy or are in early pregnancy.The predicted outcome is early pregnancy loss (EPL), defined as biochemical pregnancy loss or spontaneous miscarriage.

3. Input Variables

Users are required to input the following five predictors, which were identified as the most stable and influential variables in the final model:

D-dimer: Plasma D-dimer level reflecting fibrin turnover and coagulation activation.

Fibrin degradation products (FDP): Marker of fibrinolytic activity.

Body mass index (BMI): Pre-pregnancy or early pregnancy BMI (kg/m²).

Anti-Müllerian hormone (AMH): Indicator of ovarian reserve and PCOS phenotype.

Activated partial thromboplastin time (APTT): Measure of intrinsic coagulation pathway activity.

4. Risk Calculation and Output

After entering the required variables and submitting the form, the calculator provides:

Dynamic nomogram display: An interactive nomogram illustrating the contribution of each predictor.

Total score: A composite score derived from the weighted predictors.

Predicted EPL risk (%): Individualized probability of early pregnancy loss mapped monotonically from the total score.

Visualization of key risk contributors: The top five predictors are displayed with relative contribution scores ranging from 0 to 100.

5. Model Logic

The calculator uses a static risk-mapping approach derived from the training dataset. A monotonic transformation function links the total score to the predicted EPL probability.

Only the top five predictors identified by model importance ranking are required as inputs, allowing for simplified clinical implementation while preserving predictive performance.

6. Instructions for Use

Users enter the five required variables and click the “Calculate Risk” button to obtain an individualized EPL risk estimate expressed as a percentage.

7. Disclaimer

This tool is provided for research and educational purposes only. It is not intended to provide medical advice, diagnosis, or treatment, and should not be used as the sole basis for clinical decision-making. Clinical judgment by qualified healthcare professionals remains essential.

**Notes S2.** Clinical Interpretation and Bedside Application Framework

To enhance the clinical interpretability and translational applicability of the XGBoost model, we further integrated an individualized SHAP-based explanation strategy with a simplified bedside risk stratification framework. This approach was designed to bridge the gap between nonlinear machine-learning prediction and real-world early pregnancy assessment in patients with PCOS.

A representative patient case was included to illustrate the cumulative effect of multiple borderline abnormalities. During the first-trimester assessment, the patient presented with mildly elevated D-dimer (0.80 mg/L) and FDP (5.65 mg/L), while BMI (23.10 kg/m²), APTT (27.30 s), and AMH (5.82 ng/mL) remained within or near the normal reference range. None of these variables alone reached conventional intervention thresholds. However, the XGBoost model predicted an EPL risk probability of 86.07%, and the patient subsequently experienced clinical miscarriage during follow-up.

SHAP waterfall analysis further demonstrated that D-dimer represented the dominant positive contributor to risk prediction, followed by FDP, whereas BMI exerted a smaller but consistent additive effect. In contrast, APTT contributed minimally to the final prediction. These findings suggest that early pregnancy loss risk in PCOS may arise not from a single markedly abnormal biomarker, but from the combined influence of subtle coagulation activation, metabolic burden, and endocrine background. Importantly, SHAP decomposition enabled visualization of the biological pathways driving individual risk predictions, thereby improving model transparency and clinical interpretability.

Based on these findings, we further proposed a simplified bedside application framework for preliminary clinical stratification. Clinicians may initially evaluate three core dimensions during early pregnancy assessment: (1) coagulation status (D-dimer, FDP, APTT), (2) metabolic burden (BMI), and (3) endocrine background (AMH). When computational tools are available, individualized risk probabilities can be generated directly using the XGBoost model. In resource-limited bedside settings, patients may instead be categorized into low-, intermediate-, or high-risk groups according to the overall combination pattern of these variables rather than isolated thresholds. Particular attention should be paid to the cumulative effect of multiple borderline abnormalities, even when individual markers do not exceed conventional clinical cutoffs.

This framework does not replace quantitative model prediction, but rather serves as an interpretable clinical interface to facilitate early screening, risk communication, and individualized monitoring strategies.

**Notes S3.** Outcome Imbalance Analysis

The imbalance among pregnancy outcome groups reflected the natural distribution of outcomes in this real-world retrospective PCOS cohort rather than design-related sampling bias. Because the primary prediction task focused on binary early pregnancy loss (EPL) prediction, biochemical pregnancy and clinical miscarriage were combined as the EPL endpoint to improve clinical continuity and model stability. To preserve the original outcome prevalence and maintain calibration reliability, the original cohort structure was retained without artificial re-sampling or matching procedures. Sensitivity analyses further demonstrated that the models maintained balanced discrimination performance under the imbalanced outcome distribution, with stable calibration and clinically meaningful net benefit.

**Notes S4.** Description of Medication Exposure and Coagulation Parameter Collection

In the present study, to minimize pharmacological interference with coagulation parameters, all participants were required to be free from anticoagulant or antiplatelet therapies (e.g., low-molecular-weight heparin or aspirin) at the time of enrollment. All coagulation parameters were derived from laboratory test results obtained during the first clinical visit in early pregnancy.

Regarding metformin exposure, reliable quantification was not feasible due to the lack of standardized documentation in the retrospective database (including information on initiation timing, dosage adjustments, discontinuation, and treatment adherence). Additionally, intermittent dosing was observed in some patients due to medication intolerance. Consequently, metformin was not included as a covariate in the multivariable analysis or machine learning modeling pipeline.

Future prospective studies should incorporate standardized recording of medication exposure to further clarify its impact on coagulation function and pregnancy outcomes.

B. **Supplementary figures**

Figure S1. External validation ROC curve of the XGBoost model

**Notes:** External validation was performed using an independent temporal cohort from the same center (January–June 2025) without model retraining or parameter adjustment.

Figure S2. Decision curve analysis of the XGBoost model in external validation

Notes：Net benefit was calculated across threshold probabilities to evaluate the potential clinical utility of the prediction model for early risk stratification.

Figures S3. Multivariable logistic regression analysis of EPL subtypes

Notes: Multivariable logistic regression models for biochemical pregnancy and clinical miscarriage compared with normal pregnancy. Forest plots present adjusted odds ratios (ORs) and 95% confidence intervals for coagulation-related and clinical variables. D-dimer and FDP remained independently associated with both EPL subtypes after multivariable adjustment.

Figures S4. XGBoost ROC curves for EPL subtype models

Notes: Receiver operating characteristic (ROC) curves of XGBoost models for biochemical pregnancy and clinical miscarriage. The models demonstrated good discriminative performance, with AUCs of 0.9138 and 0.9392, respectively.

Figure S5. Beside risk stratification and representative individual-level interpretation

Notes: (A)The bedside framework was developed to facilitate preliminary early-pregnancy risk assessment in patients with PCOS when full computational prediction tools are unavailable. The framework integrates coagulation status (D-dimer, FDP, APTT), metabolic burden (BMI), and endocrine background (AMH), emphasizing the cumulative effect of multiple borderline abnormalities rather than isolated threshold exceedance. This approach is intended for preliminary clinical triage and does not replace quantitative XGBoost probability prediction.

(B) The representative case illustrates how the XGBoost model identifies hidden high-risk patterns through multidimensional integration of mildly abnormal biomarkers. Although individual laboratory variables remained within or near conventional clinical reference ranges, the combined pattern of coagulation activation and metabolic background resulted in a high predicted EPL risk. SHAP decomposition demonstrated that D-dimer and FDP were the major positive contributors to risk prediction, supporting the biological relevance of coagulation-fibrinolysis dysregulation in early pregnancy loss.

FiguresS6. Multivariable logistic regression analysis of factors independently associated with early pregnancy loss.

Notes: Forest plot summarizing adjusted odds ratios (ORs) and 95% confidence intervals (CIs) for factors independently associated with early pregnancy loss (EPL) in the pooled multivariable logistic regression model.Estimates were derived from 20 multiply imputed datasets and restricted to the training cohort. Odds ratios greater than 1 indicate higher odds of EPL, whereas values less than 1 indicate lower odds. Adjusted odds ratios were estimated using a pooled multivariable logistic regression model across 20 multiply imputed datasets. All covariates shown were included simultaneously in the model. Results represent statistical associations and do not imply causality. Model discrimination in the training set yielded a macro-average area under the curve (AUC) of 0.762.

Figures S7. Feature importance ranking and selection using the Boruta algorithm for early pregnancy loss prediction.

Notes: The Boruta algorithm is a wrapper feature selection method based on random forests that identifies relevant predictors by comparing their importance to that of randomized shadow features. Importance values are standardized as Z-scores. This procedure aims to identify a robust set of candidate predictors for subsequent modeling and does not estimate effect sizes or imply causal relationships. Box plots display the distribution of feature importance scores (Z-scores) for each candidate predictor generated by the Boruta algorithm. Variables are classified as *Confirmed*, *Rejected*, or *Shadow* features based on comparisons with randomly permuted shadow variables. Predictors whose importance consistently exceeded the maximum shadow importance were retained as confirmed features. This feature selection process was performed in the training set prior to model development. Confirmed features were subsequently subjected to LASSO regression for further dimensionality reduction.

C. **Supplementary tables**

**Table S1.** Definition and handling of candidate predictors

| **Variable** | **Category** | **Definition** | **Unit** | **Data type** | **Handling in analysis** |
| --- | --- | --- | --- | --- | --- |
| Age | Demographic | Maternal age at first clinical visit | years | Continuous | Original |
| Pre-pregnancy BMI | Clinical | Body mass index prior to pregnancy | kg/m² | Continuous | Original |
| History of adverse pregnancy | Clinical | Previous history of miscarriage or adverse pregnancy outcomes | Yes/No | Binary | Original |
| AMH | Endocrine | Serum anti-Müllerian hormone level prior to pregnancy | ng/mL | Continuous | Multiple imputation (PMM) |
| Testosterone | Endocrine | Serum total testosterone level | nmol/L | Continuous | Multiple imputation (PMM) |
| Insulin resistance | Metabolic | Clinically defined insulin resistance based on medical records | Yes/No | Binary | Original |
| Metabolic abnormality | Metabolic | Based on hyperlipidemia status in clinical records | Yes/No | Binary | Original |
| Thyroid dysfunction | Endocrine | Defined based on the presence or absence of abnormal TPOAb and TSH results in clinical records | Yes/No | Binary | Original |
| Thrombin time (TT) | Coagulation | Thrombin time | s | Continuous | Original |
| Activated partial thromboplastin time (APTT) | Coagulation | Activated partial thromboplastin time | s | Continuous | Original |
| Prothrombin activity | Coagulation | Prothrombin activity | % | Continuous | Original |
| Fibrinogen (FIB) | Coagulation | Plasma fibrinogen level | g/L | Continuous | Original |
| D-dimer | Coagulation | Fibrin degradation marker reflecting thrombus formation | µg/mL | Continuous | Original |
| Fibrin degradation products (FDP) | Coagulation | Fibrin degradation products | mg/L | Continuous | Original |
| Antithrombin III (AT-III) | Coagulation | Antithrombin III activity | % | Continuous | Original |
| Factor VIII | Coagulation | Coagulation factor VIII activity | % | Continuous | Original |
| Anti-Xa activity | Coagulation | Anti-Xa activity | IU/mL | Continuous | Original |

Notes:All candidate predictors included in this study were derived from routine clinical records and laboratory measurements, covering demographic characteristics, reproductive history, endocrine parameters, metabolic status, thyroid-related status, and coagulation–fibrinolysis markers. Detailed definitions, units, and analytical handling of each variable are provided in Supplementary Table S1.It should be noted that insulin resistance and metabolic abnormality were recorded in the original clinical database as binary indicators based on clinical assessment, without corresponding continuous measurements (e.g., insulin, glucose, or lipid levels). Therefore, these variables were incorporated as covariates reflecting overall metabolic status rather than modeled as continuous predictors.Thyroid-related status was defined based on the combined assessment of TPOAb and TSH results in clinical records and is presented as “thyroid dysfunction” to improve clinical interpretability and consistency.

**Tables S2.** Univariable logistic regression analyses of factors associated with biochemical pregnancy versus normal pregnancy among women with polycystic ovary syndrome.

| **Variable** | **Overall P value** | **Crude OR (95% CI)** | **P value** |
| --- | --- | --- | --- |
| D-Dimer | <0.001^***^ | 1.62 (1.47–1.78) | <0.001^***^ |
| FDP | <0.001^***^ | 1.24 (1.18–1.31) | <0.001^***^ |
| APTT | <0.001^***^ | 1.09 (1.06–1.12) | <0.001^***^ |
| FIB | <0.001^***^ | 1.62 (1.37–1.92) | <0.001^***^ |
| Factor VIII | <0.001^***^ | 1.00 (1.00–1.01) | <0.001^***^ |
| BMI | <0.001^***^ | 1.50 (1.26–1.78) | <0.001^***^ |
| Testosterone | <0.001^***^ | 1.22 (1.10–1.34) | <0.001^***^ |
| Anti–Xa activity | 0.0025^**^ | 18.53 (2.82–121.80) | 0.0024^**^ |
| AMH | <0.001^***^ | 1.05 (1.01–1.09) | 0.0058 |
| TT | 0.3113 | 1.08 (0.98–1.20) | 0.1298 |
| Age | 0.0013^**^ | 0.98 (0.95–1.01) | 0.1363 |
| AT3 | 0.8458 | 1.01 (0.99–1.02) | 0.4185 |
| PT | 0.342 | 1.00 (0.99–1.02) | 0.4918 |
| Dyslipidemia: Yes | 0.0266^*^ | 0.62 (0.41–0.93) | 0.0223^*^ |
| Insulin resistance：Yes | <0.001^***^ | 0.76 (0.53–1.08) | 0.1254 |
| Thyroid dysfunction: Yes | 0.0807 | 1.34 (0.88–2.05) | 0.1732 |
| Adverse obstetric history: Yes | 0.1912 | 1.16 (0.81–1.66) | 0.4229 |

Notes: Univariable logistic regression models were used to evaluate crude associations between individual predictors and pregnancy outcome. Results are presented as crude odds ratios (ORs) with 95% confidence intervals (CIs). No covariate adjustment was performed in these analyses. Variables reaching statistical significance in univariable analyses were considered for inclusion in subsequent multivariable models. All tests were two-sided, and a P value < 0.05 was considered statistically significant. *P<0.05, ^**^P<0.01, ^***^P<0.001

**Table S3.** Missing data profile and handling strategy for candidate predictors in the study cohort

| **Variable** | **Unit** | **Missing, n (%)** | **Handling strategy** |
| --- | --- | --- | --- |
| Outcome | | | |
| Early pregnancy loss (EPL) | - | 0 (0.0) | Not applicable |
| Primary predictors (coagulation parameters) | | | |
| D-dimer | µg/mL | 0 (0.0) | Complete case |
| Fibrin degradation products (FDP) | mg/L | 0 (0.0) | Complete case |
| Activated partial thromboplastin time (APTT) | s | 0 (0.0) | Complete case |
| Thrombin time (TT) | s | 0 (0.0) | Complete case |
| Fibrinogen (FIB) | g/L | 0 (0.0) | Complete case |
| Prothrombin activity | % | 0 (0.0) | Complete case |
| Antithrombin III (AT-III) | % | 0 (0.0) | Complete case |
| Factor VIII | % | 0 (0.0) | Complete case |
| Anti-Xa activity | IU/mL | 0 (0.0) | Complete case |
| Demographic and clinical characteristics | | | |
| Age | years | 0 (0.0) | Complete case |
| Pre-pregnancy body mass index (BMI) | kg/m² | 0 (0.0) | Complete case |
| History of adverse pregnancy | - | 0 (0.0) | Complete case |
| Endocrine and metabolic covariates | | | |
| Anti-Müllerian hormone (AMH) | ng/mL | 15.0 | Multiple imputation (MICE) |
| Testosterone | nmol/L | 18.0 | Multiple imputation (MICE) |
| Thyroid peroxidase antibody (TPOAb) positivity | - | 10.4 | Multiple imputation (MICE) |
| Metabolic abnormality | - | 17.0 | Multiple imputation (MICE) |
| Insulin resistance (HOMA-IR) | - | 0 (0.0) | Complete case |

Abbreviations: EPL, early pregnancy loss; FDP, fibrin degradation products; APTT, activated partial thromboplastin time; TT, thrombin time; FIB, fibrinogen; AT-III, antithrombin III; BMI, body mass index; AMH, anti-Müllerian hormone; TPOAb, thyroid peroxidase antibody; MICE, multiple imputation by chained equations.

Notes:

1. Missingness was limited to selected covariates, with all proportions below 20%.

2. No missing data were observed in the outcome or in coagulation-related variables, which were the primary predictors of interest.

3. Multiple imputation (m = 20) was performed for incomplete covariates using chained equations, with predictive mean matching for continuous variables and logistic regression for binary variables.

4. All subsequent analyses were conducted on imputed datasets, and estimates were combined using Rubin’s rules.
